# Effects of brain endurance training on cognitive function, physical performance and perceived exertion in healthy adults: a systematic review and three-level meta-analysis

**DOI:** 10.3389/fphys.2026.1923855

**Published:** 2026-10-05

**Authors:** Xin Zheng, Yu’an Sun, Ziren Zhao, Chao Wang, Zhangyuting Shang, Yixun Xie, Kai Xu, Kaixiang Zhou, Olivier Girard

**Affiliations:** 1 College of Physical Education and Health Science, Chongqing Normal University, Chongqing, China; 2 College of Sports and Health, Chengdu University of Traditional Chinese Medicine, Chengdu, Sichuan, China; 3 Sports Coaching College, Beijing Sport University, Beijing, China; 4 College of Physical Education and Health Management, Chongqing University of Education, Chongqing, China; 5 Department of Physical Education, Tongji University, Shanghai, China; 6 School of Athletic Performance, Shanghai University of Sport, Shanghai, China; 7 School of Human Sciences (Exercise and Sport Science), University of Western Australia, Perth, WA, Australia

**Keywords:** brain endurance training, cognitive function, healthy adults, perceived exertion, physical performance

## Abstract

**Background:**

Mental fatigue impairs cognitive function and physical performance in healthy adults. Brain endurance training (BET) has emerged as a strategy to enhance resistance to mental fatigue and improve both cognitive and physical outcomes; however, its effects have not been comprehensively synthesized.

**Objective:**

To systematically assess the effects of BET on cognitive function, physical performance, and perceived exertion in healthy adults.

**Methods:**

Searches were conducted in PubMed, Web of Science, Embase, Cochrane Library, and SPORTDiscus on September 9, 2025. Data (mean ± standard deviation and sample size) were extracted, and pooled effects were calculated as Hedges’g using random-effects meta-analysis models. Subgroup and meta-regression analyses were performed to explore potential moderators.

**Results:**

Eleven studies (13 trials; n=441 participants) were included. BET significantly improved cognitive function (g = 0.64, 95% CI [0.24, 1.04], P = 0.006) and physical performance (g = 0.48, 95% CI [0.33, 0.62], p<0.001), whereas no statistically significant effect was observed for perceived exertion (g = −0.01, 95% CI [−0.25, 0.23]). Effects on cognitive function and physical performance appeared larger under mental-fatigue than non-fatigued testing conditions. Evidence for other potential moderators was inconclusive, and intervention duration was not significantly associated with any outcome. The certainty of evidence was low for cognitive function and physical performance and moderate for perceived exertion.

**Conclusion:**

BET was associated with improvements in cognitive function and physical performance in healthy adults, with effects appearing larger under mental-fatigue testing conditions. BET had no statistically significant effect on perceived exertion. Given the limited number of studies and low certainty of evidence for the primary performance outcomes, these findings should be interpreted cautiously.

## 1 Introduction

Mental fatigue is a psychobiological state induced by prolonged and demanding cognitive activity. It is characterized by subjective feelings of mental tiredness accompanied by impaired cognitive function and, in some contexts, reduced physical performance (Marcora et al., 2009; Sun et al., 2021; Yuan et al., 2023; Karim et al., 2024). Mental fatigue has been consistently associated with impairments in executive functions (e.g., inhibitory control, sustained attention, and working memory), as well as increased perceived exertion during subsequent exercise performed at a given workload (Boksem et al., 2005; Boksem and Tops, 2008).

Perceived exertion is a central regulatory percept that governs pacing, persistence, and volitional task termination (Hampson et al., 2001; Brick et al., 2016). Even small increases in perceived exertion can meaningfully impair performance (Eston, 2012; Abbiss et al., 2015), particularly during intense exercise where sustained self-regulation and repeated decision-making under rising effort are key determinants of success (Pageaux, 2014). Consequently, interventions designed to increase tolerance to cognitive strain and reduce perceived exertion during exercise may represent a promising strategy for improving physical performance and maintaining cognitive function under stress.

Brain endurance training (BET) has emerged as one such strategy. BET involves repeated exposure to concurrent cognitive and physical demands to improve effort regulation during exercise. In 2015, Marcora et al. (Staiano et al., 2015) introduced the concept of BET, demonstrating that combining cognitive tasks with endurance training prolonged time to exhaustion compared to endurance training alone (Staiano et al., 2015). These findings support the hypothesis that targeting central/perceptual determinants of performance may induce adaptations beyond those achieved through physical training alone. Subsequent studies have suggested that BET could improve physical performance (e.g., endurance, muscular strength, and sport-specific skills) and cognitive functions relevant for performance, including reaction time, decision-making under pressure, and sustained concentration (Díaz-García et al., 2024; Dallaway et al., 2024; Díaz-García et al., 2025). Several trials have also reported reductions in ratings of perceived exertion (RPE) during exercise following BET, consistent with the premise that BET may modify how effort is experienced and regulated at a given workload (Díaz-García et al., 2023; Díaz-García et al., 2024; Staiano et al., 2025; Varesco et al., 2025). Collectively, these findings position BET as a mechanism-informed strategy for mitigating the performance-limiting effects of mental fatigue.

Despite this promise, the available evidence remains heterogeneous and, in several respects, inconsistent. Effects on perceived exertion, cognitive outcomes, or physical performance have not been uniformly observed, and null findings reported alongside positive results (Dallaway et al., 2021; Staiano et al., 2022; Díaz-García et al., 2023; Dallaway et al., 2023; de Lima‐Junior et al., 2023; Staiano et al., 2023; Díaz-García et al., 2024; Dallaway et al., 2024; Díaz-García et al., 2025; Staiano et al., 2025; Varesco et al., 2025). For example, a 6-week dual-task BET protocol reduced RPE and improved psychomotor vigilance and shot accuracy in padel players (Díaz-García et al., 2023), whereas a 5-week sequential-task BET protocol did not elicit similar benefits in healthy undergraduates (Dallaway et al., 2024). This inconsistency may reflect differences in participant characteristics (e.g., age, training status), BET protocols (e.g., task type, duration, and frequency), or outcome assessment methods.

An additional unresolved question concerns testing condition – specifically, whether BET effects differ when performance is assessed in a mental-fatigue versus non-fatigued testing conditions. From a mechanistic perspective, this distinction is non-trivial. If BET primarily enhances resistance to mental fatigue, its benefits should be most apparent when performance is tested after experimentally induced mental fatigue. Conversely, effects may be attenuated when testing occurs without prior cognitive strain. Current evidence on this issue is mixed. Some studies report improvements in physical performance (e.g., muscle strength) under both conditions (Dallaway et al., 2021; Dallaway et al., 2023), whereas another found that BET enhanced muscle strength only when participants were tested after experimentally induced mental fatigue using a Stroop task (Díaz-García et al., 2024). Other work has reported no improvement in specialized fencing performance under either testing condition (Varesco et al., 2025). Together, these observations suggest that testing condition may represent a meaningful moderator, but the direction and magnitude of its influence remain uncertain.

Beyond conceptual uncertainty, methodological considerations further complicate the synthesis of the BET literature. Several BET trials report multiple outcomes within the same sample (e.g., several physical performance endpoints, multiple cognitive measures, repeated perceived exertion assessments), resulting in statistically dependent effect sizes. Conventional meta-analytic approaches that assume independence may either discard valuable information (by selecting a single outcome per study) or produce biased precision estimates (by treating correlated effects as independent). A three-level meta-analysis likely provides a more appropriate framework by explicitly modeling sampling variance (level 1), within-study variance across multiple outcomes (level 2), and between-study variance (level 3), thereby enabling inclusion of multiple correlated outcomes while maintaining valid statistical inference.

To our knowledge, a comprehensive synthesis of the effects of BET on cognitive function, physical performance, and perceived exertion in healthy adults is lacking. Therefore, we conducted a systematic review and three-level meta-analysis of peer-reviewed studies to quantify these effects. The primary objective was to estimate the pooled effects of BET on cognitive function, physical performance, and perceived exertion. The secondary objective was to quantify heterogeneity and examine prespecified moderators, including participant characteristics, BET protocol features, and testing conditions (mental-fatigue versus non-fatigued testing conditions), to inform the rational design of BET interventions in future research.

## 2 Methods

### 2.1 Registration and reporting

This systematic review was conducted in accordance with the Preferred Reporting Items for Systematic Reviews and Meta-Analyses (PRISMA) guidelines(**Supplementary Table 1**) (Chandler et al., 2019). The protocol was prospectively registered in PROSPERO (ID: CRD420251068086) on 06 Jun 2025.

### 2.2 Search strategy

Two researchers (X.Z. and Z.Z.) independently searched five electronic databases (PubMed, Web of Science, Embase, Cochrane Library, and SPORTDiscus). Additional records were identified by manually screening reference lists of included studies and relevant BET review articles. The search scope was limited to studies published between 1 January 2015 and 9 September 2025, as the BET concept was first introduced in 2015 (Staiano et al., 2015). The full search strategy is provided in the (**Supplementary Table 3**).

### 2.3 Eligibility criteria

#### 2.3.1 Inclusion criteria

Studies were included if they met the following criteria: 1) Population (P): healthy adults aged ≥18 years; 2) Intervention (I): BET, defined as a training protocol combining physical exercise with cognitively demanding tasks (e.g., Stroop, flanker, and n-back) designed to induce mental fatigue and enhance tolerance to cognitive load; 3) Comparison (C): a matched physical training protocol without concurrent cognitive tasks; 4) Outcomes (O): at least one quantifiable measure of cognitive function (e.g., executive function, vigilance), physical performance (e.g., V̇O_2max_, endurance, strength, sport-specific performance), or perceived exertion (rating of perceived exertion scale [RPE]); 5) Study design (S): randomized controlled trials or controlled trials with a comparator group.

#### 2.3.2 Exclusion criteria

Studies were excluded if they: 1) Included participants< 18 years or clinical populations (e.g., neurological, psychiatric, or cardiometabolic conditions); 2) Examined acute BET protocols (i.e., a single session without repeated training over days or weeks); 3) Lacked a control group or used a control group without a training intervention; 4) Were conference abstracts, reviews, or non-English publications.

### 2.4 Study selection, data extraction and outcomes

Two independent reviewers (X.Z. and C.W.) used EndNote X9 to remove duplicate records. They screened titles and abstracts against the inclusion and exclusion criteria, and subsequently assessed the full texts of potentially eligible studies.

Two reviewers (X.Z. and K.X.) independently extracted data using a predesigned Excel form. Extracted information included author details, sample size, participant characteristics, BET protocols, and outcome measures. For each study, post-intervention means and standard deviations were extracted. If required data were unavailable, corresponding authors were contacted. If no response was received and results were presented graphically, values were extracted using GetData Graph Digitizer. A third reviewer (K.Z.) resolved any discrepancies.

### 2.5 Risk of bias and methodological characteristics assessment

The Cochrane Risk of Bias Tool 2 (RoB 2) (Higgins et al., 2011; Sterne et al., 2019) and Non-Randomized Studies of Interventions (ROBINS-I) (Morgan et al., 2018) were employed to assess methodological quality for randomized and nonrandomized studies, respectively. Two reviewers (X.Z. and Z.S.) independently conducted assessments, with a third reviewer (K.X.) arbitrating disagreements. These evaluations informed the Grading of Recommendations Assessment, Development, and Evaluation (GRADE) (West et al., 2002; Guyatt et al., 2011) assessment for the certainty of evidence. Additionally, the physiotherapy evidence database (PEDro) (de Morton, 2009) scale developed for rating the validity of interventions through critical appraisal, provides a comprehensive measure of methodological quality of included studies and across 11 criteria, with total scores of 0 to 10 (≥6: high quality; 4–6: moderate; and ≤3: low quality).

### 2.6 Statistical analysis

#### 2.6.1 Data synthesis and effect measures

For the primary analysis, the postintervention mean, SD, and sample size were extracted for each intervention and control group (Higgins et al., 2019). Because the included studies assessed outcomes using different measurement scales, standardized mean differences (SMD) were calculated to enable pooling across studies. Considering the relatively small sample sizes of the included studies, SMD were corrected for small-sample bias and expressed as Hedges’g (Hedges, 1981). Hedges’g was calculated from postintervention values as follows (Borenstein et al., 2009):


$$g=\left(\left(M_{\left(post,1\right)}-M_{\left(post,2\right)}\right)/SD_{\left(pooled,post\right)}\right)\times \left(1-3/\left(4\left(n_{1}+n_{2}-2\right)-1\right)\right)$$

where Mpost,1 and Mpost,2 represent the postintervention means of the intervention and control groups, respectively; n1 and n2 represent the corresponding sample sizes; and SD_pooled_,post represents the pooled postintervention SD, calculated as follows (Borenstein et al., 2009):


$$SD_{\left(pooled,post\right)}=\sqrt{\left(\left(\left(n_{1}-1\right)SD_{\left(post,1\right)}^{2}+\left(n_{2}-1\right)SD_{\left(post,2\right)}^{2}\right)/\left(n_{1}+n_{2}-2\right)\right)}$$

To ensure a consistent interpretation of effect direction across outcomes, effect sizes for measures in which lower values represented better outcomes were multiplied by −1 before synthesis, except for Perceived Exertion (RPE). Consequently, positive Hedges’g values consistently indicated effects favoring BET, whereas negative values indicated effects favoring the control condition; for RPE, lower values indicated more favorable outcomes.

As a sensitivity analysis, effect sizes were additionally calculated using pre- to postintervention change scores to examine whether the pooled estimates were affected by the method used to calculate effect sizes. The mean change (M_change_) for each group was calculated as (Becker, 1988):


$$M_{\left(change\right)}=M_{\left(post\right)}-M_{\left(pre\right)}$$

and the SD of the change (SD_change_) was calculated as (Cumpston et al., 2019):


$$SD_{\left(change\right)}=\sqrt{\left(SD_{\left(pre\right)}^{2}+SD_{\left(post\right)}^{2}-2rSD_{\left(pre\right)}SD_{\left(post\right)}\right)}$$

where Mpre and Mpost represent the pre- and postintervention means, respectively; SD_pre_ and SD_post_ represent the corresponding SD; and r represents the correlation between pre- and postintervention measurements (Cumpston et al., 2019). Because pre–post correlations were rarely reported, r = 0.6 (Cumpston et al., 2019)was used as the primary assumption, with alternative values examined in sensitivity analyses. Change-score Hedges’g was subsequently calculated using the pooled SD of the change scores.

Considering the relatively small sample sizes of most BET studies, Hedges’g correction was applied to the SMD to correct for small-sample bias (Cohen, 2013). Hedges’g values were classified as trivial (<0.2), small (0.2–0.5), moderate (>0.5–0.8), and large (>0.8) (DerSimonian and Kacker, 2007).

#### 2.6.2 Meta-analysis and heterogeneity

A three-level random-effects meta-analysis was conducted in R using the metafor package to pool Hedges’g while accounting for the dependency of multiple effect sizes within studies (Van den Noortgate et al., 2013). An approximate variance–covariance matrix was constructed using impute_covariance_matrix, assuming a within-study correlation of r = 0.6, to account for correlated effect sizes derived from the same study (Pustejovsky and Tipton, 2022). The three-level model was fitted using rma.mv, with random effects specified at the effect-size and study levels, thereby partitioning the variance into sampling variance (level 1), within-study variance (level 2), and between-study variance (level 3) (Cheung, 2019). Cluster-robust inference with small-sample adjustment was subsequently applied at the study level using the robust function with the clubSandwich method (Hedges et al., 2010).

95% CIs were calculated using the Knapp–Hartung adjustment with dfs = ”contain.” Prediction intervals (PI) were derived from the t-distribution for analyses including > 5 studies (Nakagawa et al., 2017). Heterogeneity was quantified using *I²* statistics, with values of 0%–25%, 25%–50%, 50%–75%, and 75%–100% interpreted as low, moderate, substantial, and considerable heterogeneity, respectively (Quintana, 2023). Between-study heterogeneity was additionally characterized using the estimated variance components (τ²) (Cumpston et al., 2019).

Potentially influential effect sizes were identified using Cook’s distances, with values exceeding three times the mean Cook’s distance considered influential (Aguinis et al., 2013). Because highly influential observations may exert disproportionate effects on pooled estimates and variance components, these effect sizes were excluded from the primary analyses. The overall three-level meta-analysis, subgroup analyses, and meta-regression analyses were subsequently re-estimated using the remaining data. Analyses retaining these influential effect sizes were conducted as sensitivity analyses to evaluate the impact of their exclusion.

### 2.7 Subgroup and meta-regression analysis

To explore heterogeneity and potential moderating factors, subgroup analyses were conducted for categorical variables and meta-regression analyses for continuous variables when sufficient studies were available (Deeks et al., 2019). Subgroup analyses examined test conditions (mental-fatigue vs non-fatigued testing conditions), training status (Trained vs untrained), BET Type (dual-task vs sequential-task design), intervention frequency (3, 4, or 5 sessions/week), and cognitive intervention characteristics (multi-task vs single-task paradigm). Outcome-specific subgroup analyses were also performed. Cognitive Function was categorized as Executive Function or Vigilance, whereas Physical Performance was categorized as Aerobic Capacity, Muscular Endurance, or Sport-Specific Performance.

Meta-regression analyses (Deeks et al., 2019) were conducted to examine the linear association between BET intervention duration (weeks) and effect sizes for Cognitive Function, Physical Performance, and Perceived Exertion.

### 2.8 Risk of publication bias and sensitivity analysis

Publication bias was assessed using contour-enhanced funnel plots and Egger-type analyses were exploratory because of the limited number of independent trials and dependent effect sizes. A P value >.05 was considered to indicate no statistically significant evidence of publication bias (Schünemann et al., 2019; Jukic et al., 2021).

Sensitivity analyses were conducted to assess the robustness of the pooled estimates. These included (1) performing leave-one-out analyses to evaluate the influence of individual studies on the overall estimates; (2) varying the assumed within-study correlation coefficient (r = 0.4, 0.6, and 0.8) used to construct the covariance matrix; (3) varying the assumed pre–post correlation coefficient (r = 0.4, 0.6, and 0.8) used to calculate change-score effect sizes; (4) restricting the analyses to randomized controlled trials by excluding nonrandomized studies; and (5) restricting the analyses to studies using passive control conditions. Sensitivity analyses restricted to active control conditions were not conducted because of the limited number of eligible studies.

### 2.9 Certainty of the evidence

The certainty of evidence was evaluated using the GRADE framework and categorized as high, moderate, low, or very low (Guyatt et al., 2011). Assessments were initially conducted by one reviewer (X.Z.) and independently verified by a second reviewer (Y.S.).

The certainty of evidence was evaluated across five domains: risk of bias, inconsistency, indirectness, imprecision, and publication bias. Evidence was downgraded by one level for each domain judged to have serious concerns. Risk of bias was considered serious when more than 50% of the included studies were judged to be at high risk of bias; inconsistency was considered serious when *I²* exceeded 50%; indirectness was assessed according to the applicability of the population, intervention, comparator, and outcomes to the review question; imprecision was judged according to whether the 95% CI crossed prespecified decision-relevant thresholds; and publication bias was considered serious when Egger’s regression test indicated significant small-study effects (P< 0.05) or when substantial funnel plot asymmetry was observed.

## 3 Results

The study selection process is shown in **Figure 1**. A total of 1768 records were retrieved (PubMed n=91, Web of Science n=1121, Embase n=197, SPORTDiscus n=17, Cochrane Library n=342). After title and abstract screening, 1755 studies were excluded. Of the 13 full-text articles assessed for eligibility, two were excluded, leaving 11 studies for inclusion. Two of these studies (Staiano et al., 2023; Dallaway et al., 2024) each included two independent randomized controlled trials, resulting in a total of 13 trials contributing to the quantitative synthesis (**Figure 1**).

**Figure 1:**
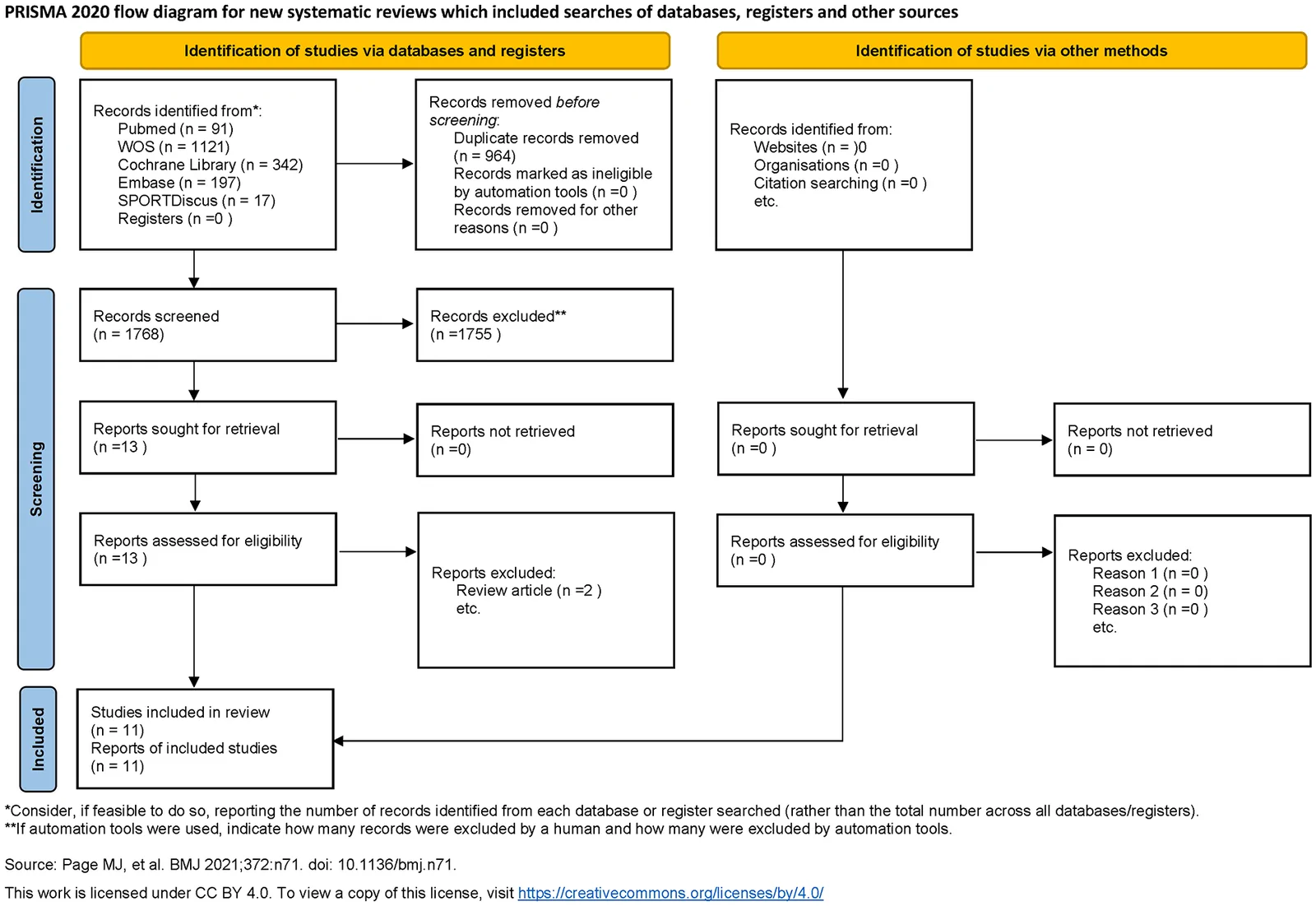
Flowchart of the selection and inclusion process, following the PRISMA statement.

### 3.1 Participant characteristics

The review included 441 healthy adults aged 18–78 years. One study did not report sex (Díaz-García et al., 2023). Of the total sample, 157 were untrained and 284 were trained athletes (e.g., cyclists, football players, fencers, and runners) (**Table 1**).

**Table 1:** Characteristics of the included independent trials (n = 13).

Study	Participants	Sample size	BET Type	Dose	BET protocol	Comparator
Varesco et al. (2025)	Elite youth épée fencers; age 18 ± 1 y	n=19; BET = 11; CON = 8	Dual-task design	5 weeks; 3–4 BET sessions/week	Cycling and fencing training combined with PVT-B and Stroop tasks; dual-task and intermixed BET sessions were performed before, during, or after fencing training.	Habitual fencing training only
Díaz-García et al. (2023)	Amateur padel players; age 28.71 ± 7.68 y	n=61; BET = 30; CON = 31	Dual-task design	6 weeks; 3 sessions/week	Padel warm-up, technical/tactical drills and simulated games interleaved with 4-min Stroop bouts (20 min cognitive load/session).	Same padel training with rest instead of Stroop
Díaz-García et al. (2024)	Resistance-trained adults; age 29.42 ± 10.06 y	n=91; BET = 46; CON = 45	Dual-task design	6 weeks; 5 sessions/week	Chest press and squat jump training with 3-min Stroop tasks interspersed between exercise sets.	Same resistance training with rest instead of Stroop
Díaz-García et al. (2025)	Sedentary older women; age 65–78 y	n=16; BET = 8; CON = 8	Sequential-task design	8 weeks; 3 sessions/week	20-min Stroop task performed before 20-min resistance exercise and 25-min endurance walking.	Passive control
Staiano et al. (2022)	Professional football players; age 22.4 ± 4.3 y	n=25; BET = 13; CON = 12	Sequential-task design	4 weeks; 4–5 BET sessions/week	Football training followed by 20–30 min of cognitive response-inhibition tasks (Flanker, Go/No-Go, AX-CPT).	Football training followed by neutral audio
Staiano et al. (2023), Study 1	Road cyclists; age 29 ± 5 y	n=26; BET = 13; CON = 13	Sequential-task design	6 weeks; 5 sessions/week	Cycling training followed by 30–60 min of cognitive response-inhibition tasks (Flanker, Go/No-Go, AX-CPT).	Cycling training followed by neutral music/audio
Staiano et al. (2023), Study 2	Male road cyclists; age 25 ± 4 y	n=24; BET = 13; CON = 11	Sequential-task design	6 weeks; 5 sessions/week	Cycling training followed by 30 min of cognitive response-inhibition tasks (Flanker, Go/No-Go, AX-CPT).	Cycling training followed by neutral music/audio
Staiano et al. (2025)	Professional male soccer players; age 26.32 ± 8.41 y	n=31; BET = 15; CON = 16	Dual-task design	6 weeks; 3 sessions/week	Physical training with 24 min of cognitive tasks intermixed within the training session (Flanker, Go/No-Go, AX-CPT).	Same physical training without cognitive tasks
de Lima‐Junior et al. (2023)	Amateur trained runners; age 23.4 ± 2.9 y	n=30; BET = 15; CON = 15	Dual-task design	12 weeks; 3 sessions/week	Treadmill endurance training performed simultaneously with a Stroop word–color task; 20–40 min/session.	Matched endurance training alone
Dallaway et al. (2021)	Healthy undergraduate students; age 20 ± 2 y	n=36; BET = 18; CON = 18	Dual-task design	6 weeks; 4 sessions/week	Submaximal rhythmic handgrip training with concurrent 2-back and Stroop cognitive tasks.	Matched handgrip training without high cognitive load
Dallaway et al. (2023)	Healthy undergraduate students; age 20 ± 2 y	n=24; BET = 12; CON = 12	Sequential-task	5 weeks; 4 sessions/week	20-min cognitive training (2-back/Stroop) performed before submaximal rhythmic handgrip training (~30% MVC).	Matched handgrip training without prior cognitive task
Dallaway et al. (2024), Study 1	Healthy undergraduate students; age 23 ± 5 y	n=29; BET = 14; CON = 15	Dual-task design	4 weeks; 3 sessions/week	Calisthenic training (press-ups, wall sit and plank) interleaved with 3-min high-load cognitive tasks.	Matched calisthenic training with low cognitive load
Dallaway et al. (2024), Study 2	Healthy undergraduate student-athletes; age 21 ± 2 y	n=29; BET = 15; CON = 14	Dual-task design	4 weeks; 3 sessions/week	Calisthenic training preceded/intermixed with cognitive tasks including 2-back, switching-stop visual task, MSIT and time-load dual n-back task.	Matched calisthenic training with rest instead of cognitive tasks

### 3.2 Intervention protocols

Intervention duration ranged from 3 to 12 weeks, with training frequencies of 3–5 sessions per week. Cognitive-task sessions typically lasted 20–30 minutes. Two studies combined soccer-specific training with cognitive tasks (Staiano et al., 2022; Staiano et al., 2025), two combined cycling training with cognitive tasks (Staiano et al., 2023), one involved resistance training (Díaz-García et al., 2024), and two incorporated comprehensive fitness training (e.g., press-ups, wall sit, and plank) with cognitive tasks (Dallaway et al., 2024). Two studies used rhythmic handgrip training (Dallaway et al., 2021; Dallaway et al., 2023), while other studies combined cognitive tasks with cycling training plus fencing-specific training (Varesco et al., 2025), resistance and walking training (Díaz-García et al., 2025), padel-specific training (Díaz-García et al., 2023), or running training (de Lima‐Junior et al., 2023).

The implementation of BET consisted of two main approaches. Five studies adopted a sequential-task design, in which cognitive tasks were performed before and after physical training (Staiano et al., 2022; Dallaway et al., 2023; Staiano et al., 2023; Díaz-García et al., 2025). Eight studies used a dual-task design, where cognitive tasks were performed concurrently with exercise or recovery periods (Dallaway et al., 2021; Díaz-García et al., 2023; de Lima‐Junior et al., 2023; Díaz-García et al., 2024; Dallaway et al., 2024; Staiano et al., 2025; Varesco et al., 2025). Three types of cognitive tasks were employed: response inhibition (e.g., Stroop, Flanker, Go/No-Go, AX-CPT), working memory updating (e.g., n-back), and vigilance (PVT). Cognitive load was manipulated in three ways: extending task duration [3 studies (de Lima‐Junior et al., 2023; Staiano et al., 2023);], increasing task difficulty [4 studies; (i.e., adding distractors or shortening stimulus intervals) (Dallaway et al., 2021; Dallaway et al., 2023; Staiano et al., 2023; Staiano et al., 2025)], or maintaining a fixed task difficulty [6 studies (Staiano et al., 2022; Díaz-García et al., 2023; Díaz-García et al., 2024; Dallaway et al., 2024; Díaz-García et al., 2025; Varesco et al., 2025);]. Seven studies employed a single cognitive task (e.g., Stroop task) (Dallaway et al., 2021; Díaz-García et al., 2023; Dallaway et al., 2023; de Lima‐Junior et al., 2023; Díaz-García et al., 2024; Díaz-García et al., 2025; Varesco et al., 2025), while four employed multiple-task paradigms (e.g., Flanker, Go/No-Go, AX-CPT) (Staiano et al., 2022; Staiano et al., 2023; Dallaway et al., 2024; Staiano et al., 2025). Control conditions involved either simple cognitive tasks (e.g., consistent Stroop task, 0-back task) (Staiano et al., 2022; Staiano et al., 2023; Dallaway et al., 2024) or quiet rest (Dallaway et al., 2021; Díaz-García et al., 2023; Dallaway et al., 2023; de Lima‐Junior et al., 2023; Díaz-García et al., 2024; Díaz-García et al., 2025; Staiano et al., 2025; Varesco et al., 2025).

### 3.3 Outcome measurements

Outcomes were assessed under two conditions: non-fatigued testing condition and after mental fatigue induction. In the non-fatigued testing condition, participants completed tests such as a 5-minute time trial (TT), RPE and PVT-B accuracy. These assessments were then repeated following mental fatigue induction using tasks such as Stroop or n-back. A schematic illustration of the testing conditions is provided in **Figure 2**.

**Figure 2:**
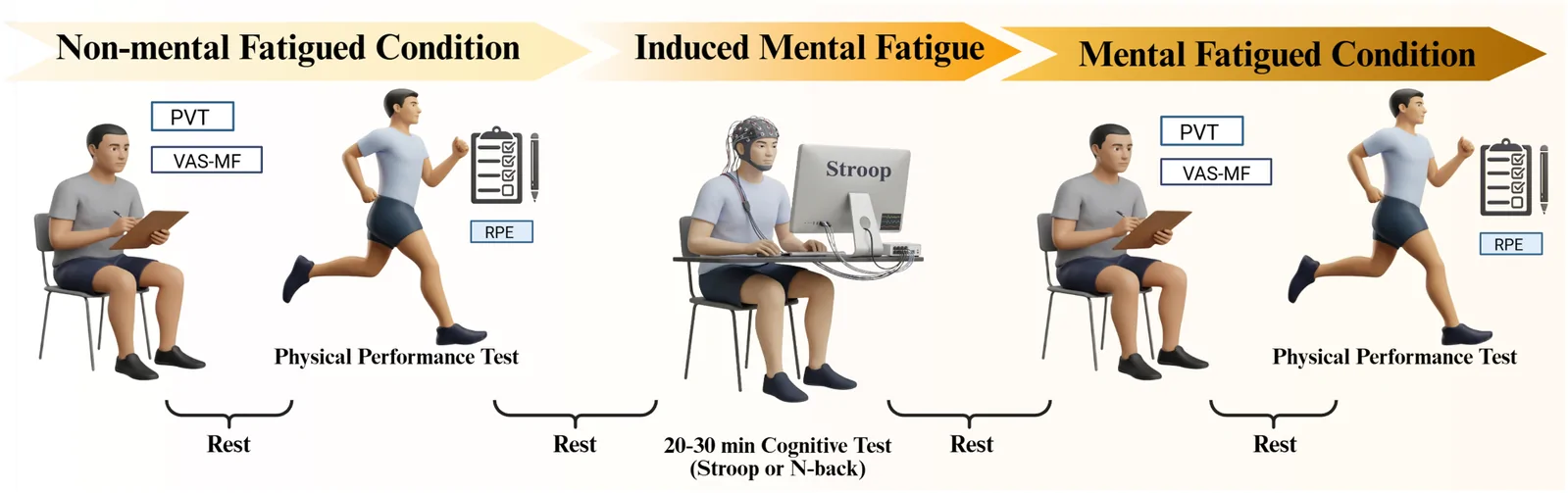
BET test flowchart.

Seven studies (Dallaway et al., 2023; de Lima‐Junior et al., 2023; Staiano et al., 2023; Dallaway et al., 2024; Díaz-García et al., 2025) evaluated perceived exertion, cognitive function, and physical performance. Perceived exertion was measured using RPE. Cognitive function was evaluated via Stroop task reaction time (Stroop RT) and accuracy (Stroop Accuracy), PVT-B reaction time (PVT-B RT), and 2-back task reaction time (2-back RT). Physical performance outcomes included aerobic measures (e.g., V̇O_2__peak_, time to exhaustion [TTE], and 6-min walk distance) and muscular endurance (e.g., chair-stand repetitions and arm-curl repetitions). One study (Dallaway et al., 2021) evaluated perceived exertion (i.e., RPE) and cognitive function (i.e., 2-back accuracy) only. Five studies (Staiano et al., 2022; Díaz-García et al., 2023; Díaz-García et al., 2024; Staiano et al., 2025; Varesco et al., 2025) evaluated cognitive function and physical performance. Cognitive outcomes included Stroop RT, Stroop Accuracy, PVT-B RT, and PVT-B lapses. Physical performance measures included aerobic capacity (e.g., repeated sprint ability), muscular endurance (e.g., chest press and squat jump), and sport-specific performance (e.g., the fencing specialized performance test, padel shot speed, and padel shot accuracy). Additional physiological outcomes included root mean square of the successive differences, heart rate, pupil diameter, and changes in oxygenated and deoxygenated hemoglobin (**Supplementary Table 2**).

### 3.4 Primary analysis

The meta-analysis found a significant benefit of BET interventions compared with control conditions for improving different measures of cognitive function (g = 0.64, 95% CI [0.24 to1.04], I²_2_ = 80.65%, I²_3_ = 1.75%, P = 0.006, PI [-1.51, 3.11], Low GRADE) (**Figure 3**; **Supplementary Table 6**). The meta-analysis found a significant benefit of BET interventions compared with control conditions for improving different measures of physical performance (g = 0.48, 95% CI [0.33 to 0.62], I²_2_ = 80.7%, I²_3_ =0.00%, P<0.001, PI [-1.06, 2.02], Low GRADE) (**Figure 4**; **Supplementary Table 6**). The meta-analysis found no significant benefit of BET interventions compared with control conditions for improving different measures of perceived exertion (g =-0.01, 95% CI [-0.25 to 0.23], I²_2_ = 18.35%, I²_3_ = 0.00%, P = 0.92, PI [-0.54, 0.52], Moderate GRADE) (**Figure 5**; **Supplementary Table 6**).

**Figure 3:**
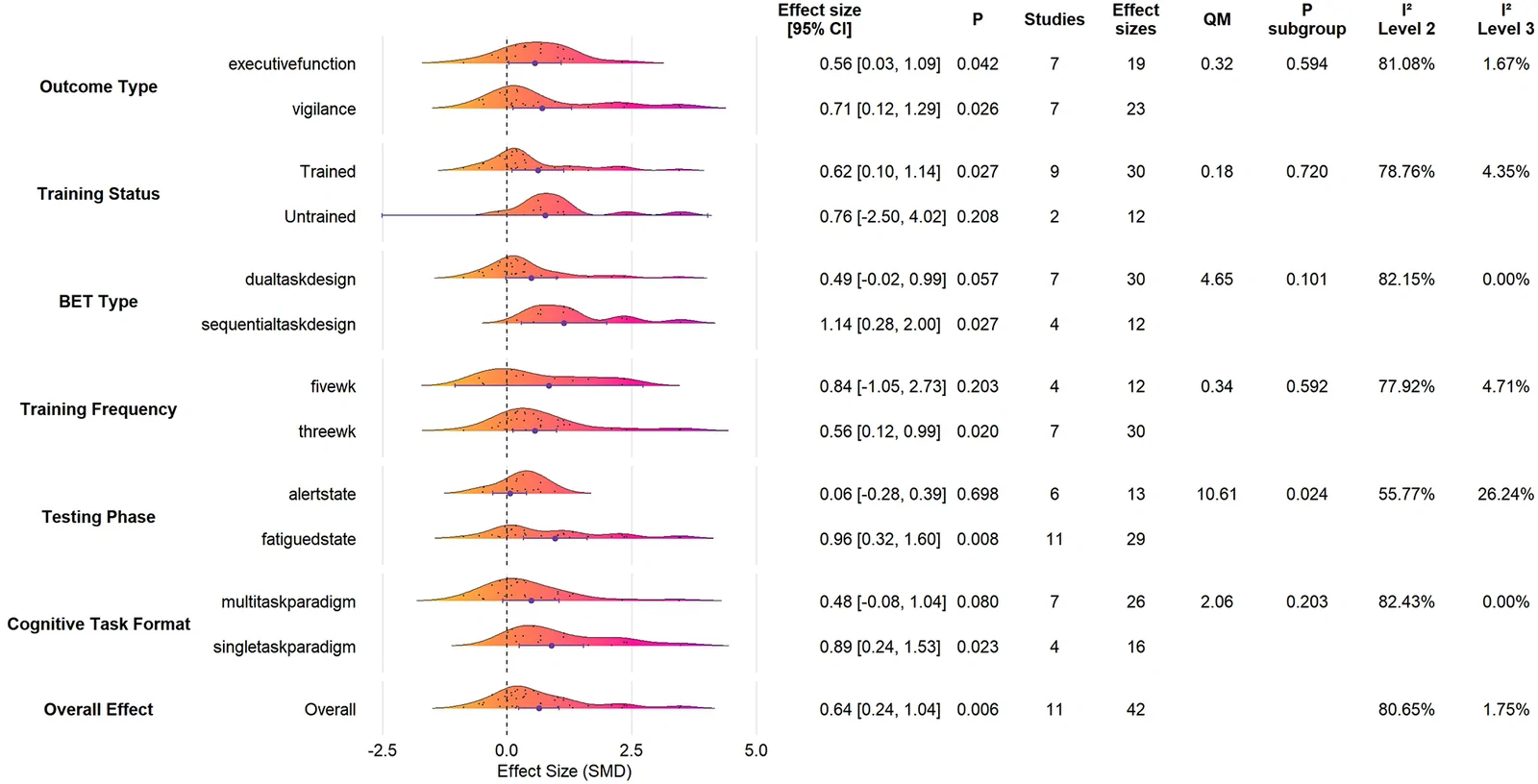
Effect of BET on cognitive function.

**Figure 4:**
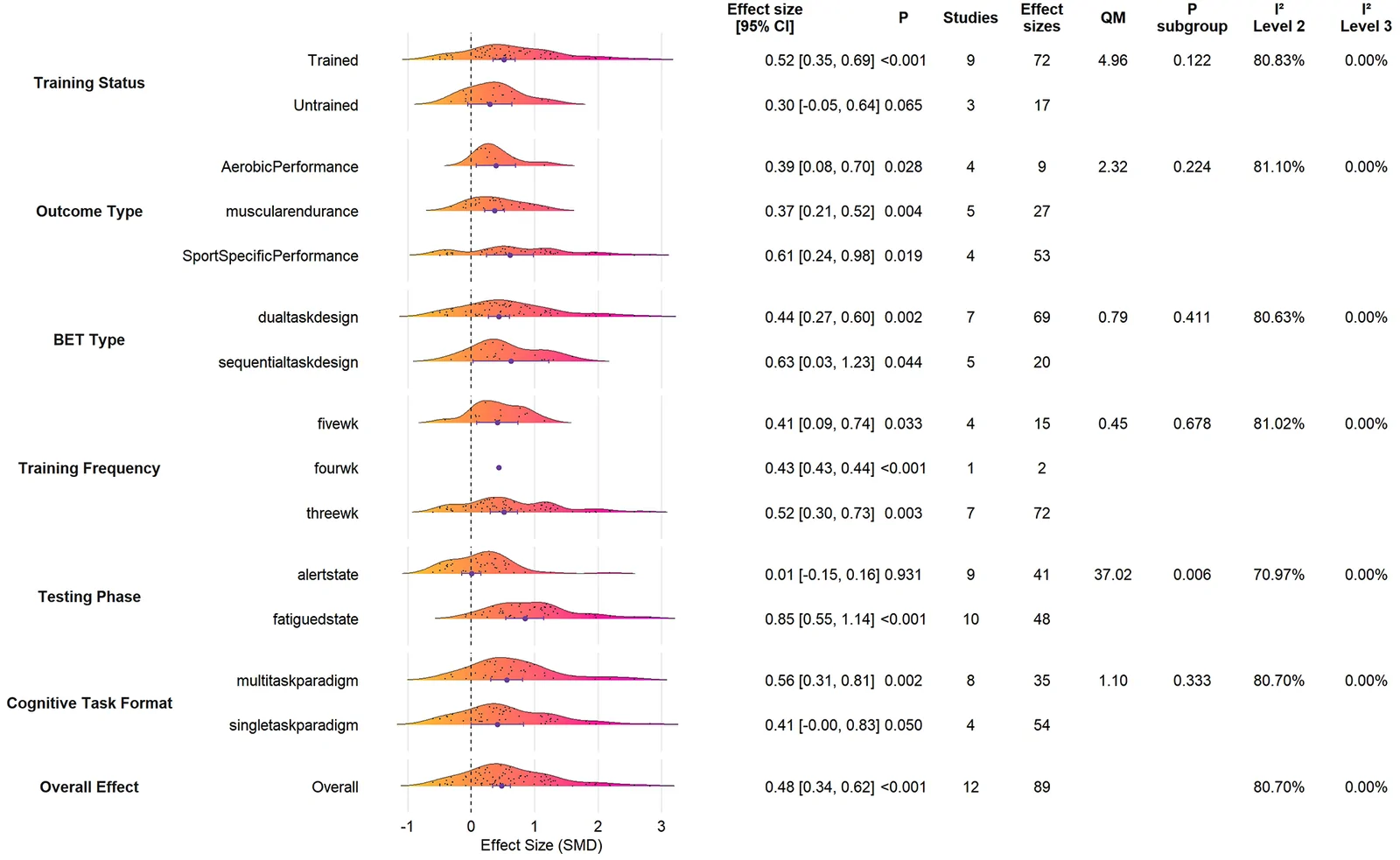
Effect of BET on physical performance.

**Figure 5:**
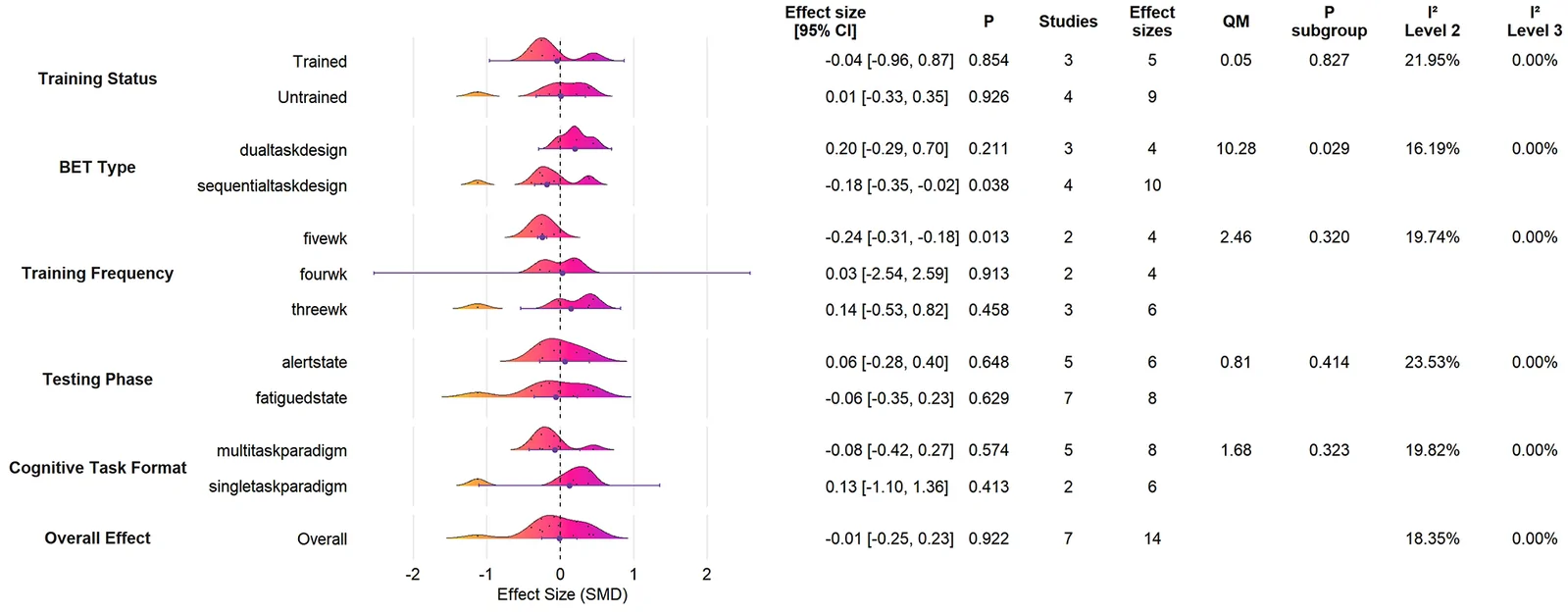
Effect of BET on perceived exertion.

### 3.5 Moderator analysis

#### 3.5.1 Potential moderators on cognitive function

Regarding Test conditions, BET was associated with a significant improvement in cognitive performance under mental-fatigue testing conditions (g = 0.96, 95% CI [0.32, 1.60], P = 0.008), whereas no significant effect was observed under non-fatigued testing conditions (g = 0.06, 95% CI [−0.28, 0.39], P = 0.70), with a significant between-subgroup difference (P = 0.024). Thus, effects appeared larger under Fatigued than Non-fatigued testing conditions. Regarding Training status, a significant improvement was observed in Trained individuals (g = 0.62, 95% CI [0.10, 1.14], P = 0.03), whereas the effect in Untrained individuals did not reach statistical significance (g = 0.76, 95% CI [−2.50, 4.02], P = 0.21). Regarding Cognitive outcome type, BET significantly improved both Executive function (g = 0.56, 95% CI [0.03, 1.09], P = 0.04) and Vigilance (g = 0.71, 95% CI [0.12, 1.29], P = 0.03). Regarding Cognitive task formats, BET significantly improved cognitive performance under the Single-task paradigm (g = 0.89, 95% CI [0.24, 1.53], P = 0.02), whereas the effect under the Multitask paradigm did not reach statistical significance (g = 0.48, 95% CI [−0.08, 1.04], P = 0.08), and the between-subgroup difference was not significant (P = 0.203). Regarding BET Type, significant improvements in cognitive performance were observed for the Sequential-task design (g = 1.14, 95% CI [0.28, 2.00], P = 0.03), whereas the effect for the Dual-task design did not reach statistical significance (g = 0.49, 95% CI [−0.02, 0.99], P = 0.06). Regarding Frequency, BET performed 3/week significantly improved cognitive performance (g = 0.56, 95% CI [0.12, 0.99], P = 0.02), whereas the effect of BET performed 5/week was not statistically significant (g = 0.84, 95% CI [−1.05, 2.73], P = 0.20) (**Figure 3**). Meta-regression analysis revealed no significant linear relationship between improvements in cognitive performance and BET intervention duration in weeks (β = −0.06, 95% CI [−0.27, 0.15], P = 0.38) (**Figure 6**).

**Figure 6:**
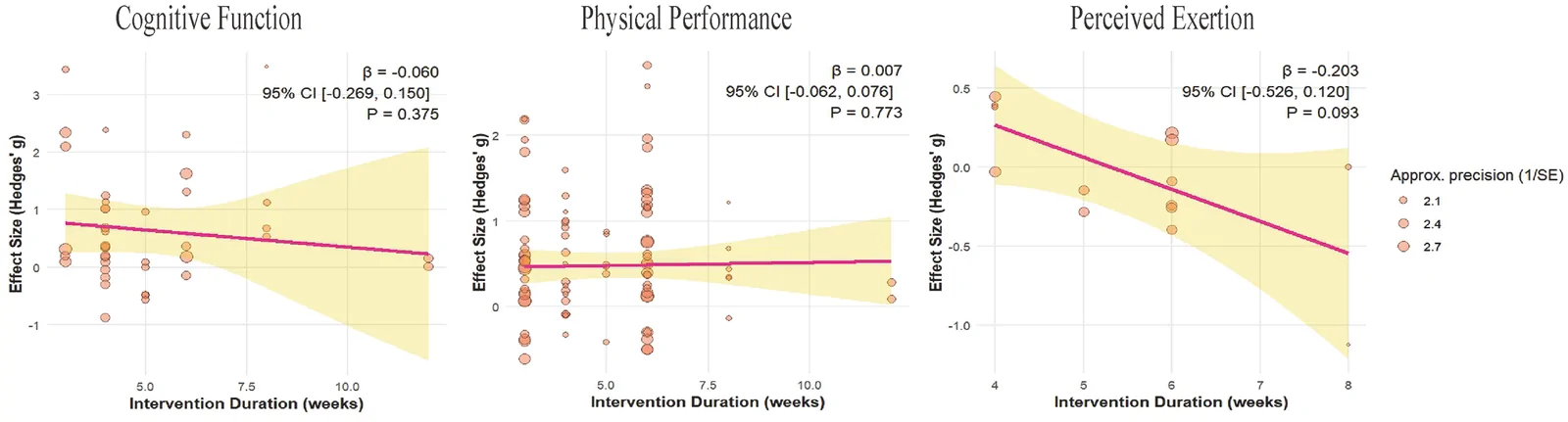
Linear meta-regression of BET intervention duration (weeks).

#### 3.5.2 Potential moderators on physical performance

Regarding Test conditions, BET was associated with a significant improvement in physical performance under mental-fatigue testing conditions (g = 0.85, 95% CI [0.55, 1.14], P< 0.001), whereas no significant effect was observed under non-fatigued testing conditions (g = 0.01, 95% CI [−0.15, 0.16], P = 0.93), with a significant between-subgroup difference (P = 0.006). Thus, effects appeared larger under Fatigued than Non-fatigued testing conditions. Regarding Training Status, a significant improvement was observed in Trained individuals (g = 0.52, 95% CI [0.35, 0.69], P< 0.001), whereas the effect in Untrained individuals did not reach statistical significance (g = 0.30, 95% CI [−0.05, 0.64], P = 0.07). Regarding Physical performance types, BET significantly improved Aerobic capacity (g = 0.39, 95% CI [0.08, 0.70], P = 0.03), Muscular endurance (g = 0.37, 95% CI [0.22, 0.52], P = 0.004), and Sport-specific performance (g = 0.61, 95% CI [0.24, 0.98], P = 0.02). Regarding BET Type, significant improvements in physical performance were observed for both Dual-task design (g = 0.44, 95% CI [0.27, 0.60], P = 0.002) and Sequential-task design (g = 0.63, 95% CI [0.03, 1.23], P = 0.04). Regarding Frequency, significant improvements in physical performance were observed for 3/week (g = 0.52, 95% CI [0.30, 0.73], P = 0.003), 4/week (g = 0.43, 95% CI [0.43, 0.44], P< 0.001), and 5/week (g = 0.41, 95% CI [0.09, 0.74], P = 0.03). Regarding Cognitive intervention characteristics, BET significantly improved physical performance under the Multi-task paradigm (g = 0.56, 95% CI [0.31, 0.81], P = 0.002), whereas the effect under the Single-task paradigm did not clearly reach statistical significance (g = 0.41, 95% CI [−0.00, 0.83], P = 0.05) (**Figure 4**). Meta-regression analysis revealed no significant linear relationship between improvements in physical performance and BET intervention duration in weeks (β = 0.01, P = 0.77) (**Figure 6**).

#### 3.5.3 Potential moderators on perceived exertion

Regarding BET Type, BET produced a significant reduction in perceived exertion under the Sequential-task design (g = −0.18, 95% CI [−0.35, −0.02], P = 0.04), whereas the effect under the Dual-task design did not reach statistical significance (g = 0.20, 95% CI [−0.29, 0.70], P = 0.21), with a significant between-subgroup difference (P = 0.029). Regarding Frequency, BET performed 5/week significantly reduced perceived exertion (g = −0.25, 95% CI [−0.31, −0.18], P = 0.013), whereas the effects of BET performed 4/week (g = 0.03, 95% CI [−2.54, 2.59], P = 0.91) and 3/week (g = 0.14, 95% CI [−0.54, 0.82], P = 0.46) were not statistically significant. Regarding Test conditions, no significant effects on perceived exertion were observed under either non-fatigued testing conditions (g = 0.06, 95% CI [−0.28, 0.40], P = 0.65) or mental-fatigue testing conditions (g = −0.06, 95% CI [−0.35, 0.23], P = 0.63). Regarding Training status, BET did not significantly affect perceived exertion in either Trained individuals (g = −0.04, 95% CI [−0.96, 0.88], P = 0.85) or Untrained individuals (g = 0.01, 95% CI [−0.33, 0.35], P = 0.93). Regarding Cognitive intervention characteristics, no significant effects on perceived exertion were observed under either the Multi-task paradigm (g = −0.08, 95% CI [−0.42, 0.27], P = 0.57) or the Single-task paradigm (g = 0.13, 95% CI [−1.10, 1.36], P = 0.41) (**Figure 5**). Meta-regression analysis revealed no significant linear relationship between changes in perceived exertion and BET intervention duration in weeks (β = −0.20, 95% CI [−0.53, 0.12], P = 0.09) (**Figure 6**).

### 3.6 Risk of bias and methodological characteristics

The risk-of-bias assessment is presented in **Supplementary Figure 1**. Twelve studies (Dallaway et al., 2021; Staiano et al., 2022; Díaz-García et al., 2023; Dallaway et al., 2023; de Lima‐Junior et al., 2023; Staiano et al., 2023; Díaz-García et al., 2024; Dallaway et al., 2024; Díaz-García et al., 2025; Staiano et al., 2025) did not report sufficient details regarding randomization methods and allocation concealment, resulting in “some concerns” for the randomization process. Another study was rated as “some concerns” due to participant dropout and failure to employ intention-to-treat analyses (Staiano et al., 2022). Blinding of outcome assessors was rarely reported; only Jesús Díaz-García et al. (2023) explicitly disclosed blinding procedures. The remaining eleven studies (Dallaway et al., 2021; Staiano et al., 2022; Díaz-García et al., 2023; Dallaway et al., 2023; Staiano et al., 2023; Díaz-García et al., 2024; Dallaway et al., 2024; Díaz-García et al., 2025; Staiano et al., 2025) were rated as “some concerns” for this domain due to insufficient reporting. Finally, nearly all studies were assessed as “some concerns” because of the absence of prospective trial registration. One non-randomized controlled study (Varesco et al., 2025) was assessed as having a high risk of bias (**Supplementary Figure 2**). The mean PEDro score was 5.6 (**Supplementary Table 4**).

### 3.7 Risk of publication bias

Given the limited number of studies and the presence of dependent effect sizes, publication-bias analyses were considered exploratory. Exploratory multilevel Egger-type regression indicated statistically significant small-study effects for Cognitive Function (P = 0.03) and Physical Performance (P = 0.05), suggesting possible small-study effects rather than definitive evidence of publication bias. In contrast, the Egger-type regression did not detect statistically significant small-study effects for Perceived Exertion (P = 0.22) (**Figure 7**; **Supplementary Table 5**).

**Figure 7:**
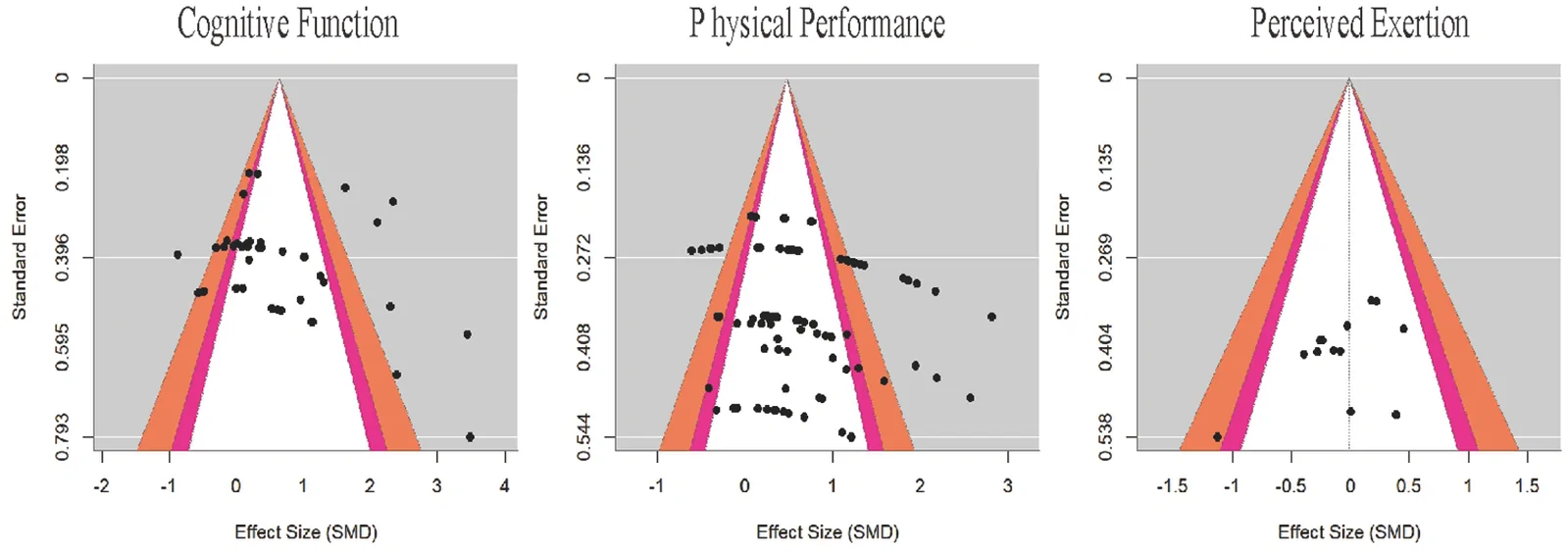
Funnel plots on cognitive function, physical performance, and perceived exertion.

### 3.8 Sensitivity analysis

Leave-one-out sensitivity analysis indicated that no single study substantially altered the overall pooled estimates. Sensitivity analyses were conducted for cognitive function, physical performance, and perceived exertion. After outlier removal, varying the assumed within-study correlation coefficient (r = 0.4–0.8) yielded pooled effects of g = 0.64–0.65 for Cognitive Function, g = 0.46–0.51 for Physical Performance, and g = −0.01 to −0.01 for Perceived Exertion. Varying the assumed pre–post correlation coefficient (r = 0.4–0.8) yielded g = 0.65–0.98, g = 0.41–0.67, and g = −0.03 to −0.01, respectively. Effects remained significant for Cognitive Function and Physical Performance but non-significant for Perceived Exertion across these correlation assumptions. After excluding non-RCT studies, the pooled effects were g = 0.73 (95% CI [0.30, 1.16], P = 0.005), g = 0.47 (95% CI [0.31, 0.63], P< 0.001), and g = −0.01 (95% CI [−0.25, 0.23], P = 0.92), respectively. When analyses were restricted to passive control conditions, the corresponding effects were g = 0.43 (95% CI [−0.05, 0.91], P = 0.07), g = 0.44 (95% CI [0.28, 0.60], P = 0.001), and g = 0.09 (95% CI [−0.33, 0.51], P = 0.53), respectively. Notably, the cognitive-function estimate was no longer statistically significant when analyses were restricted to passive control conditions. Analyses restricted to active control conditions were not conducted because of the limited number of eligible studies; therefore, potential differences between active and passive control conditions could not be fully evaluated (**Supplementary Table 7**; **Supplementary Figures S3**–**5**).

### 3.9 Adverse events and withdrawals

Seven participants withdrew due to injury (Staiano et al., 2023; Varesco et al., 2025). One additional participant withdrew because training intensity negatively affected sleep quality (Varesco et al., 2025).

## 4 Discussion

To our knowledge, this is the first meta-analysis to synthesize the effects of BET on cognitive function, physical performance, and perceived exertion in healthy adults. Overall, BET was associated with significant improvements in cognitive function and physical performance, with effects appearing larger under mental-fatigue testing conditions. BET had no statistically significant effect on perceived exertion. Subgroup analyses suggested that testing condition may be associated with differences in BET effects. Given the low certainty of evidence for cognitive function and physical performance, these findings should be interpreted cautiously. These findings may inform the refinement of BET protocols and highlight the need for greater standardization in future intervention and evaluation designs.

The present findings suggest that BET is associated with improvements in cognitive function, including executive function and vigilance, with larger effects appearing under mental-fatigue testing conditions. A hypothesis-generating explanation is that BET improves prefrontal cortex (PFC) regulation, thereby increasing neural efficiency during cognitive processing. Dallaway et al., 2021; Dallaway et al. (2023) reported that a 6-week BET intervention was accompanied by higher PFC oxygenation responses during endurance training compared with control training. Additionally, BET may facilitate cognitive processing by modulating connectivity within and between executive-control networks (e.g., central executive network) and attentional networks (e.g., dorsal attention network) (Fassbender et al., 2009; Esposito et al., 2014; Pageaux et al., 2014; Holroyd, 2015; Gergelyfi et al., 2021; Wiehler et al., 2022). Supporting this interpretation, one study showed that after five weeks of BET, reaction time, lapses, and perceived fatigue induced by a fatiguing task remained stable in the BET group, whereas impairments were evident in the control group (Varesco et al., 2025). Improved neural efficiency may enable athletes to allocate cognitive resources more effectively during demanding tasks or sustain performance during prolonged exercise (e.g., endurance running). Similarly, another study reported improvements in reaction speed, decision-making, inhibitory control, and concentration following BET in athletes (Staiano et al., 2022). Direct evidence supporting these neural mechanisms remains limited. Future studies incorporating neurophysiological assessments (e.g., fMRI/EEG) are needed to directly examine the neural adaptations associated with BET.

A key finding was that the effects of BET on cognitive function appeared larger under mental-fatigue than non-fatigued testing conditions. Mental fatigue is known to impair sustained attention and executive control, leading to slower reaction times and increased attentional lapses (Boksem et al., 2005; Stafylidis et al., 2025). BET may enhance top-down prefrontal regulation of goal maintenance and inhibitory control while improving the allocation of attentional resources under high cognitive load. This may enable individuals to maintain cognitive performance during mental fatigue. This interpretation is consistent with prior evidence; for instance, Staiano et al. (2025) reported faster PVT responses in the BET group under mental fatigue, alongside a progressive attenuation in the detrimental effects of Stroop-induced fatigue on passing and shooting performance by week 6. Likewise, Varesco et al. (2025) observed that Stroop-induced slowing of PVT reaction time and increases in attention lapses occurred only in the control group after five weeks of training. Nevertheless, the observed subgroup difference may be partly confounded by outcome type, assessment timing, task similarity, near-transfer effects, task familiarity, or repeated exposure to the same cognitive task. Therefore, the larger effects observed under mental-fatigue conditions should not be interpreted as definitive evidence that testing condition modifies BET efficacy. Other examined intervention characteristics, including cognitive task format, BET Type, frequency, and intervention duration, did not provide clear evidence of moderation, although the limited number of studies reduces the precision of these analyses.

The effect size for vigilance improvement (g = 0.71) was numerically slightly larger than that for executive function (g = 0.56). One possibility is that BET tasks closely engage processes related to sustained attention and resistance to distraction, leading to shorter transfer distances between training and outcome measures. In contrast, executive function is a broader construct comprising multiple cognitive components, which may not be fully targeted by the training stimuli. Several studies (e.g., working memory training) show that cognitive training yields significant improvements in near-transfer tasks (Diamond and Ling, 2016; Simons et al., 2016), but these effects are largely confined to the trained task or closely related tasks (Melby-Lervåg and Hulme, 2013). Accordingly, gains from executive-function training are largely task-specific, with consistent near-transfer effects but limited and inconsistent far transfer across tasks and cognitive domains (Beauchamp et al., 2016; Simons et al., 2016). Dallaway et al. (2024) similarly reported that the cognitive benefits of BET are predominantly confined to near-transfer tasks. Current evidence, therefore, remains insufficient to support far-transfer effects of BET. Practice effects may also contribute to observed cognitive improvements; when BET participants repeatedly perform tasks such as the Stroop test, post-intervention differences may partly reflect greater task familiarity rather than BET-specific cognitive adaptation.

Another finding is that BET was associated with improvements in aerobic capacity, muscular endurance, and sport-specific performance in healthy adults. Several factors may contribute to these observed effects. First, repeated BET may enhance tolerance to mental fatigue and improve effort regulation during exercise (Marcora et al., 2014). Several studies have shown lower RPE at equivalent physiological intensities following BET compared with control training (Staiano et al., 2023; Díaz-García et al., 2025). Reduced perceived effort may enable athletes to push closer to their physiological limits, thereby extending time to exhaustion (TTE) or achieving a greater workload in time-based tests. However, the pooled effect of BET on perceived exertion was not statistically significant, indicating that changes in perceived exertion cannot presently be considered an established explanation for the observed improvements in physical performance. For example, Neil Dallaway et al. (2023) found that after five weeks of BET, RPE did not differ significantly between groups, yet the BET group showed superior endurance during a maximum grip strength task. Second, BET may help attenuate the detrimental effects of mental fatigue on neuromuscular performance during prolonged exercise. One study reported maintained or improved bench press and squat jump performance after fatigue-inducing protocols in the BET group, whereas performance declined in the control group (Díaz-García et al., 2024). This could be relevant for athletes required to execute repeated high-intensity efforts during competition, such as soccer and basketball players. Finally, BET may help maintain cognitive engagement during physically demanding exercise. Training under simultaneous cognitive and physical stress may improve the ability to maintain focus and decision-making under fatigue. Such adaptations may contribute to sport-specific performance under physiological stress. For example, BET has been shown to significantly improve reaction agility and decision-making ability in soccer players, both of which are relevant to match performance (Staiano et al., 2022; Roelands and Bogataj, 2024).

Subgroup analyses indicated that the effects of BET on physical performance appeared larger under mental-fatigue testing conditions. One possible explanation is that BET may help maintain higher PFC oxygenation during mental fatigue. Related physiological evidence from Dallaway et al. (2021) reported no significant post-intervention changes in HR, whereas HRV increased significantly in the BET group, suggesting reduced sympathetic activation and enhanced parasympathetic modulation. Such adaptations may help preserve executive control during mental fatigue and thereby support physical performance. Nevertheless, these physiological observations provide only indirect support for this mechanism, and the subgroup contrast may also reflect task-related factors rather than BET-specific adaptations.

Subgroup analyses also showed that the effects of BET on physical performance appeared larger in trained than in untrained individuals, with a moderate and significant effect observed in trained individuals but a smaller, non-significant effect in untrained individuals. One possible explanation is that trained individuals, who are more familiar with testing procedures and technical movements, may more readily translate improvements in effort regulation and attention control into measurable performance gains. In contrast, lower baseline physical fitness in untrained individuals may limit the extent to which such adaptations are reflected in performance outcomes. For example, Schaefer et al. (Schaefer and Scornaienchi, 2020) found that table tennis players had lower dual-task costs than untrained individuals when returning balls while performing an auditory 3-back task, suggesting that trained individuals may be better able to maintain motor performance under cognitive load. Given the limited number of studies involving untrained individuals, this subgroup pattern should be regarded as exploratory. Additional RCT are needed to clarify the effects of BET in this population.

Although the pooled analyses indicated improvements in both cognitive and physical performance, some participants withdrew due to excessive training intensity. This highlights the importance of individualization and safety considerations when designing BET interventions, particularly for untrained individuals. The effective integration of cognitive and physical training components within BET requires further exploration. Different sequencing strategies (e.g., simultaneous integration with exercise tasks, cognitive training performed prior to exercise, interleaving cognitive tasks during rest intervals within exercise sessions, or implementing cognitive tasks following exercise) may produce distinct physical and cognitive outcomes. For instance, cognitive training performed prior to exercise may enhance focus and executive control during subsequent physical training, while implementing cognitive tasks following exercise may facilitate cognitive recovery after physical exertion. Furthermore, factors such as the duration and complexity of cognitive tasks within BET and the type of physical training employed (localized vs. whole-body exercise) may also influence the magnitude and nature of the observed benefits. Several methodological considerations further temper interpretation of the findings. Sensitivity analyses generally produced similar effect estimates across alternative correlation assumptions and after exclusion of the non-randomized study; however, the cognitive-function effect was no longer statistically significant when analyses were restricted to passive control conditions. Because too few studies used active control conditions, an active-control-only analysis could not be performed, and the possibility that control type influenced effect estimates cannot be excluded. In addition, given the small number of studies and the presence of dependent effect sizes, Egger-type regression analyses should be regarded as exploratory rather than definitive evidence for or against publication bias.

### 4.1 Limitations and implications for future research

This meta-analysis has several limitations. The relatively small number of included studies and limited sample sizes may reduce statistical power and increase the risk of type I and II errors. The predominance of short-term interventions (3–12 weeks) also limits conclusions about long-term adaptation or retention effects. The variability in cognitive task types, training modalities, and mental fatigue induction protocols may introduce methodological heterogeneity that is not fully captured by subgroup analyses. The high proportion of studies rated as “some concerns” in risk-of-bias assessment (e.g., lack of pre-registration, unclear allocation concealment) may also affect internal validity. The reliance on laboratory-based cognitive tasks potentially limits ecological validity for real-world performance. Finally, the fact that only a small number of research groups have investigated BET restricts the generalizability of the current results.

Future studies should employ multi-arm study designs that include combined cognitive and physical training, cognitive-only training, physical-only training, and a control group to better differentiate the contributions of each intervention component. Further studies should explore the optimal characteristics of combined interventions, focusing on duration, frequency, type, and combination mode. Investigating the physiological mechanisms underlying BET-related improvements, using neuroimaging and molecular markers, would also provide valuable insight. Comprehensive neuropsychological assessments are needed to identify which cognitive domains benefit most from combined interventions. To distinguish learning effects from genuine synergistic adaptations, future studies should also examine transfer effects, particularly the distinction between near and far transfer. Finally, studies should consider potential attentional resource conflicts during dual-task activities, as the complexity of physical tasks may either enhance or interfere with cognitive training outcomes.

## 5 Conclusion

BET was associated with improvements in cognitive function and physical performance in healthy adults, with effects appearing larger under mental-fatigue testing conditions. BET had no statistically significant effect on perceived exertion. Evidence for other potential moderators remains inconclusive, and the findings should be interpreted cautiously given the limited number of studies and low certainty of evidence.

## 6 Practical applications

The present findings suggest that BET may be a useful complementary strategy for improving cognitive function and physical performance in healthy adults, with effects appearing larger under mental-fatigue testing conditions. For coaches and strength and conditioning practitioners, BET could be considered as an adjunct to existing training programs aimed at maintaining performance under cognitive or physical demands. BET should not replace traditional physical training but may be integrated cautiously by combining exercise with cognitive tasks such as Stroop, Flanker, n-back, or vigilance-based tasks. Practitioners should individualize cognitive-task difficulty, exercise intensity, and overall training load, particularly given the limited evidence regarding optimal BET protocols. As BET showed no statistically significant pooled effect on perceived exertion, it should not be assumed to reduce perceived effort during exercise.
