# Supplementary material for: Effects of brain endurance training on cognitive function, physical performance and perceived exertion in healthy adults: a systematic review and three-level meta-analysis
Source: Front Physiol. 2026 Oct 5;17:1923855. doi: 10.3389/fphys.2026.1923855 (PMC13678844; doi:10.3389/fphys.2026.1923855)
Supplement: Supplementary file 1 [file DataSheet1.docx]

Supplementary Material

**Effects of Brain Endurance Training** **on** **Cognitive Function, Physical Performance and** **Perceived Exertion** **in Healthy Adults: A Systematic Review and Three-Level Meta-Analysis**

Xin Zheng^1†^; Yu'an Sun^2†^; Ziren Zhao^1†^; Chao Wang^1^; Zhangyuting Shang^3,4^; Yixun Xie^5^; Kai Xu^6^; Kaixiang Zhou^1*^; Olivier Girard^7^

**Author Affiliations:**

^1^ College of Physical Education and Health Science, Chongqing Normal University, Chongqing, China;

^2^College of Sports and Health, Chengdu University of Traditional Chinese Medicine, Chengdu, Sichuan, China;

^3^ Sports Coaching College, Beijing Sport University, Beijing, China;

^4^ College of Physical Education and Health Management, Chongqing University of Education, Chongqing, China;

^5^ Department of Physical Education, Tongji University, Shanghai, China

^6^ School of Athletic Performance, Shanghai University of Sport, Shanghai, China

^7^ School of Human Sciences (Exercise and Sport Science), University of Western Australia, Perth, Western Australia, Australia

^†^Xin Zheng, Yu'an Sun and Ziren Zhao contributed equally to this work.

**Corresponding Author:**

Kaixiang Zhou, PhD, College of Physical Education and Health Science, Chongqing Normal University, No. 37, University Town Middle Road, Shapingba District, Chongqing, China; e-mail address: [20230102@cqnu.edu.cn](mailto:20230102@cqnu.edu.cn)

**Table S1 PRISMA 2020 checklist**

| - **Section and Topic** | - **Item #** | - **Checklist item** | - **Location where item is reported (on page)** |
| --- | --- | --- | --- |
| - **TITLE** | | |  |
| - Title | - 1 | - Identify the report as a systematic review. | - P1 |
| - **ABSTRACT** | | |  |
| - Abstract | - 2 | - See the PRISMA 2020 for Abstracts checklist. | - P1-2 |
| - **INTRODUCTION** | | |  |
| - Rationale | - 3 | - Describe the rationale for the review in the context of existing knowledge. | - P3-5 |
| - Objectives | - 4 | - Provide an explicit statement of the objective(s) or question(s) the review addresses. | - P3-5 |
| - **METHODS** | | |  |
| - Eligibility criteria | - 5 | - Specify the inclusion and exclusion criteria for the review and how studies were grouped for the syntheses. | - P6 |
| - Information sources | - 6 | - Specify all databases, registers, websites, organisations, reference lists and other sources searched or consulted to identify studies. Specify the date when each source was last searched or consulted. | - P6 |
| - Search strategy | - 7 | - Present the full search strategies for all databases, registers and websites, including any filters and limits used. | - P6 |
| - Selection process | - 8 | - Specify the methods used to decide whether a study met the inclusion criteria of the review, including how many reviewers screened each record and each report retrieved, whether they worked independently, and if applicable, details of automation tools used in the process. | - P6 |
| - Data collection process | - 9 | - Specify the methods used to collect data from reports, including how many reviewers collected data from each report, whether they worked independently, any processes for obtaining or confirming data from study investigators, and if applicable, details of automation tools used in the process. | - P6-7 |
| - Data items | - 10a | - List and define all outcomes for which data were sought. Specify whether all results that were compatible with each outcome domain in each study were sought (e.g. for all measures, time points, analyses), and if not, the methods used to decide which results to collect. | - P7 |
|  | - 10b | - List and define all other variables for which data were sought (e.g. participant and intervention characteristics, funding sources). Describe any assumptions made about any missing or unclear information. | - P7 |
| - Study risk of bias assessment | - 11 | - Specify the methods used to assess risk of bias in the included studies, including details of the tool(s) used, how many reviewers assessed each study and whether they worked independently, and if applicable, details of automation tools used in the process. | - P7 |
| - Effect measures | - 12 | - Specify for each outcome the effect measure(s) (e.g. risk ratio, mean difference) used in the synthesis or presentation of results. | - P7-8 |
| - Synthesis methods | - 13a | - Describe the processes used to decide which studies were eligible for each synthesis (e.g. tabulating the study intervention characteristics and comparing against the planned groups for each synthesis (item #5)). | - P8-11 |
|  | - 13b | - Describe any methods required to prepare the data for presentation or synthesis, such as handling of missing summary statistics, or data conversions. | - P8-11 |
|  | - 13c | - Describe any methods used to tabulate or visually display results of individual studies and syntheses. | - P8-11 |
|  | - 13d | - Describe any methods used to synthesize results and provide a rationale for the choice(s). If meta-analysis was performed, describe the model(s), method(s) to identify the presence and extent of statistical heterogeneity, and software package(s) used. | - P8-11 |
|  | - 13e | - Describe any methods used to explore possible causes of heterogeneity among study results (e.g. subgroup analysis, meta-regression). | - P8-11 |
|  | - 13f | - Describe any sensitivity analyses conducted to assess robustness of the synthesized results. | - P8-11 |
| - Reporting bias assessment | - 14 | - Describe any methods used to assess risk of bias due to missing results in a synthesis (arising from reporting biases). | - P8-11 |
| - Certainty assessment | - 15 | - Describe any methods used to assess certainty (or confidence) in the body of evidence for an outcome. | - P8-11 |
| - **RESULTS** | | |  |
| - Study selection | - 16a | - Describe the results of the search and selection process, from the number of records identified in the search to the number of studies included in the review, ideally using a flow diagram. | - P11 |
|  | - 16b | - Cite studies that might appear to meet the inclusion criteria, but which were excluded, and explain why they were excluded. | - P11 |
| - Study characteristics | - 17 | - Cite each included study and present its characteristics. | - P12- |
| - Risk of bias in studies | - 18 | - Present assessments of risk of bias for each included study. | - P11-16 |
| - Results of individual studies | - 19 | - For all outcomes, present, for each study: (a) summary statistics for each group (where appropriate) and (b) an effect estimate and its precision (e.g. confidence/credible interval), ideally using structured tables or plots. | - P11-16 |
| - Results of syntheses | - 20a | - For each synthesis, briefly summarise the characteristics and risk of bias among contributing studies. | - P11-16 |
|  | - 20b | - Present results of all statistical syntheses conducted. If meta-analysis was done, present for each the summary estimate and its precision (e.g. confidence/credible interval) and measures of statistical heterogeneity. If comparing groups, describe the direction of the effect. | - P11-16 |
|  | - 20c | - Present results of all investigations of possible causes of heterogeneity among study results. | - P11-16 |
|  | - 20d | - Present results of all sensitivity analyses conducted to assess the robustness of the synthesized results. | - P11-16 |
| - Reporting biases | - 21 | - Present assessments of risk of bias due to missing results (arising from reporting biases) for each synthesis assessed. | - P11-16 |
| - Certainty of evidence | - 22 | - Present assessments of certainty (or confidence) in the body of evidence for each outcome assessed. | - P11-16 |
| - **DISCUSSION** | | |  |
| - Discussion | - 23a | - Provide a general interpretation of the results in the context of other evidence. | - P16-24 |
|  | - 23b | - Discuss any limitations of the evidence included in the review. | - P16-24 |
|  | - 23c | - Discuss any limitations of the review processes used. | - P16-24 |
|  | - 23d | - Discuss implications of the results for practice, policy, and future research. | - P16-24 |
| - **OTHER INFORMATION** | | |  |
| - Registration and protocol | - 24a | - Provide registration information for the review, including register name and registration number, or state that the review was not registered. | - P25-26 |
|  | - 24b | - Indicate where the review protocol can be accessed, or state that a protocol was not prepared. | - P25-26 |
|  | - 24c | - Describe and explain any amendments to information provided at registration or in the protocol. | - N/A |
| - Support | - 25 | - Describe sources of financial or non-financial support for the review, and the role of the funders or sponsors in the review. | - P25-26 |
| - Competing interests | - 26 | - Declare any competing interests of review authors. | - P25-26 |
| - Availability of data, code and other materials | - 27 | - Report which of the following are publicly available and where they can be found: template data collection forms; data extracted from included studies; data used for all analyses; analytic code; any other materials used in the review. | - P25-26 |

**Table S2 Outcome Measures Under Non-Mental-Fatigue and Mental-Fatigue Conditions**

| Study | Outcome domain | Standardized outcome measure | Non-mental-fatigue condition | Mental-fatigue condition |
| --- | --- | --- | --- | --- |
| Varesco et al. (2025) | Cognitive function | PVT reaction time | → | → |
| Varesco et al. (2025) | Cognitive function | PVT accuracy | → | → |
| Varesco et al. (2025) | Cognitive function | PVT lapses | → | → |
| Varesco et al. (2025) | Physical performance | Fencing test performance (targets hit and execution time) | → | → |
| Díaz-García et al. (2023) | Cognitive function | PVT-B reaction time | → | ↓ |
| Díaz-García et al. (2023) | Oculomotor measure | Saccade latency | → | ↓ |
| Díaz-García et al. (2023) | Oculomotor measure | Pupil diameter | → | ↓ |
| Díaz-García et al. (2023) | Physical performance | Padel shot speed | → | ↑ |
| Díaz-García et al. (2023) | Physical performance | Padel shot accuracy | → | ↑ |
| Díaz-García et al. (2024) | Cognitive function | PVT-B reaction time | → | ↓ |
| Díaz-García et al. (2024) | Physical performance | Chest press repetitions to failure | → | ↑ |
| Díaz-García et al. (2024) | Physical performance | Squat jump repetitions to failure | → | ↑ |
| Díaz-García et al. (2024) | Physiological measure | RMSSD | → | ↓ |
| Díaz-García et al. (2025) | Perceived exertion | Rating of perceived exertion (RPE) | → | ↓ |
| Díaz-García et al. (2025) | Cognitive function | PVT-B reaction time | ↓ | ↓ |
| Díaz-García et al. (2025) | Cognitive function | Stroop accuracy | ↑ | ↑ |
| Díaz-García et al. (2025) | Physical performance | 30-s chair-stand repetitions | ↑ | ↑ |
| Díaz-García et al. (2025) | Physical performance | 30-s arm-curl repetitions | ↑ | ↑ |
| Díaz-García et al. (2025) | Physical performance | 6-min walk distance | ↑ | ↑ |
| Staiano et al. (2022) | Cognitive function | PVT lapses | → | ↓ |
| Staiano et al. (2022) | Cognitive function | PVT reaction time | → | → |
| Staiano et al. (2022) | Cognitive function | Stroop reaction time | ↓ | ↑ |
| Staiano et al. (2022) | Cognitive function | Stroop accuracy | → | → |
| Staiano et al. (2022) | Physical performance | 30–15 Intermittent Fitness Test (30–15 IFT) performance | ↑ | — |
| Staiano et al. (2022) | Physical performance | Repeated-Sprint Ability Random test (RSA random) performance | → | ↑ |
| Staiano et al. (2022) | Physical performance | Soccer-Specific Reactive Agility Test (S-RAG) performance | — | ↑ |
| Staiano et al. (2022) | Physiological measure | Blood lactate concentration at the end of the 30–15 IFT | — | → |
| Staiano et al. (2022) | Physiological measure | Heart rate at the end of the 30–15 IFT | — | → |
| Staiano et al. (2023), Study 1 | Perceived exertion | Rating of perceived exertion (RPE) | ↓ | ↓ |
| Staiano et al. (2023), Study 1 | Physical performance | Peak oxygen uptake (V̇O₂peak) | → | — |
| Staiano et al. (2023), Study 1 | Physical performance | Time to exhaustion at 80% peak power output (TTE at 80% PPO) | ↑ | — |
| Staiano et al. (2023), Study 1 | Cognitive function | Stroop reaction time | — | ↓ |
| Staiano et al. (2023), Study 1 | Cognitive function | Stroop accuracy | — | → |
| Staiano et al. (2023), Study 1 | Physical performance | Time to exhaustion at 65% peak power output (TTE at 65% PPO) | — | ↑ |
| Staiano et al. (2023), Study 2 | Perceived exertion | Rating of perceived exertion (RPE) | → | ↓ |
| Staiano et al. (2023), Study 2 | Physical performance | 5-min time-trial performance | → | — |
| Staiano et al. (2023), Study 2 | Physical performance | Peak oxygen uptake (V̇O₂peak) | → | — |
| Staiano et al. (2023), Study 2 | Cognitive function | Stroop reaction time | — | ↓ |
| Staiano et al. (2023), Study 2 | Cognitive function | Stroop accuracy | — | → |
| Staiano et al. (2023), Study 2 | Physical performance | 20-min time-trial performance | — | ↓ |
| Staiano et al. (2025) | Cognitive function | PVT-B reaction time | → | ↓ |
| Staiano et al. (2025) | Physical performance | Loughborough Soccer Passing Test (LSPT) performance | → | ↑ |
| Staiano et al. (2025) | Physical performance | Loughborough Soccer Shooting Test (LSST) performance | → | ↑ |
| de Lima-Junior et al. (2023) | Perceived exertion | Rating of perceived exertion (RPE) | ↓ | — |
| de Lima-Junior et al. (2023) | Cognitive function | Stroop reaction time | — | → |
| de Lima-Junior et al. (2023) | Cognitive function | Stroop accuracy | — | → |
| de Lima-Junior et al. (2023) | Physical performance | Maximal oxygen uptake (V̇O₂max) | → | — |
| de Lima-Junior et al. (2023) | Physical performance | Time to exhaustion (TTE) | → | — |
| de Lima-Junior et al. (2023) | Physiological/oculomotor measure | Pupil diameter | → | — |
| Dallaway et al. (2021) | Perceived exertion | Rating of perceived exertion (RPE) | → | → |
| Dallaway et al. (2021) | Physiological measure | RMSSD | ↓ | ↓ |
| Dallaway et al. (2021) | Physiological measure | Heart rate | → | → |
| Dallaway et al. (2021) | Cerebral haemodynamic measure | Change in oxyhaemoglobin concentration (ΔO₂Hb) | ↑ | ↑ |
| Dallaway et al. (2021) | Cerebral haemodynamic measure | Change in deoxyhaemoglobin concentration (ΔHHb) | → | → |
| Dallaway et al. (2021) | Cerebral haemodynamic measure | Tissue oxygenation index (TOI) | ↑ | ↑ |
| Dallaway et al. (2021) | Cerebral haemodynamic measure | Normalized total haemoglobin index (nTHI) | ↑ | ↑ |
| Dallaway et al. (2023) | Perceived exertion | Rating of perceived exertion (RPE) | → | → |
| Dallaway et al. (2023) | Physical performance | Average peak handgrip force | ↑ | ↑ |
| Dallaway et al. (2023) | Cognitive function | 2-back accuracy | — | → |
| Dallaway et al. (2023) | Physiological measure | RMSSD | → | → |
| Dallaway et al. (2023) | Physiological measure | Heart rate | → | → |
| Dallaway et al. (2023) | Cerebral haemodynamic measure | Change in oxyhaemoglobin concentration (ΔO₂Hb) | → | → |
| Dallaway et al. (2023) | Cerebral haemodynamic measure | Change in deoxyhaemoglobin concentration (ΔHHb) | → | → |
| Dallaway et al. (2023) | Cerebral haemodynamic measure | Tissue oxygenation index (TOI) | → | → |
| Dallaway et al. (2023) | Cerebral haemodynamic measure | Normalized total haemoglobin index (nTHI) | ↑ | ↑ |
| Dallaway et al. (2024), Study 1 | Perceived exertion | Rating of perceived exertion (RPE) | → | → |
| Dallaway et al. (2024), Study 1 | Physical performance | Press-up repetitions to failure | ↑ | ↑ |
| Dallaway et al. (2024), Study 1 | Physical performance | Wall-sit duration to failure | → | → |
| Dallaway et al. (2024), Study 1 | Physical performance | Plank duration to failure | → | → |
| Dallaway et al. (2024), Study 1 | Cognitive function | 2-back reaction time | — | ↓ |
| Dallaway et al. (2024), Study 1 | Cognitive function | AX-CPT reaction time | — | ↓ |
| Dallaway et al. (2024), Study 2 | Perceived exertion | Rating of perceived exertion (RPE) | — | → |
| Dallaway et al. (2024), Study 2 | Cognitive function | 4-choice reaction time | → | → |
| Dallaway et al. (2024), Study 2 | Cognitive function | Mackworth Clock Task performance | → | → |
| Dallaway et al. (2024), Study 2 | Cognitive function | Rapid Visual Information Processing task performance | → | → |
| Dallaway et al. (2024), Study 2 | Physical performance | Burpee repetitions to failure | → | ↑ |
| Dallaway et al. (2024), Study 2 | Physical performance | Jump-squat repetitions to failure | → | ↑ |
| Dallaway et al. (2024), Study 2 | Physical performance | Leg-raise repetitions to failure | → | ↑ |
| Dallaway et al. (2024), Study 2 | Physical performance | Press-up repetitions to failure | → | → |
| Dallaway et al. (2024), Study 2 | Physical performance | Plank duration to failure | → | → |
| Dallaway et al. (2024), Study 2 | Physical performance | Mountain-climber repetitions to failure | → | ↑ |
| Notes | | | | |
| *↑ indicates an increase in the reported measure; ↓ indicates a decrease in the reported measure; → indicates no clear/statistically significant change as coded in the original extraction; — indicates that the outcome was not reported for that condition. Arrow direction refers to the numerical direction of the outcome and does not by itself indicate improvement or deterioration.* | | | | |
| *Abbreviations: AX-CPT, AX Continuous Performance Task; BET, brain endurance training; LSPT, Loughborough Soccer Passing Test; LSST, Loughborough Soccer Shooting Test; MF, mental fatigue; MSIT, Multi-Source Interference Task; NMF, non-mental fatigue; PPO, peak power output; PVT, psychomotor vigilance task; PVT-B, brief psychomotor vigilance task; RMSSD, root mean square of successive differences; RPE, rating of perceived exertion; S-RAG, Soccer-Specific Reactive Agility Test; TTE, time to exhaustion; V̇O₂max, maximal oxygen uptake; V̇O₂peak, peak oxygen uptake.* | | | | |

**Table S3 Search strategy.**

| Data | Keywords | N |
| --- | --- | --- |
| Pubmed | (("brain endurance training"[Title/Abstract] OR "cognitive endurance training"[Title/Abstract] OR "mental endurance training"[Title/Abstract] OR "cognitive fatigue training"[Title/Abstract] OR "mental fatigue training"[Title/Abstract] OR (("mental fatigue"[Title/Abstract] OR "cognitive fatigue"[Title/Abstract]) AND training[Title/Abstract]))AND("Athletic Performance"[Mesh] OR "athletic performance"[Title/Abstract] OR "sports performance"[Title/Abstract] OR "sport performance"[Title/Abstract] OR "physical performance"[Title/Abstract] OR "technical performance"[Title/Abstract])AND("Athletes"[Mesh] OR athlete*[Title/Abstract] OR player*[Title/Abstract] OR sport*[Title/Abstract])) | 91 |
| Web of Science | TS= ("brain endurance training" OR "cognitive endurance training" OR "mental endurance training" OR "cognitive fatigue training" OR "mental fatigue training") AND TS=("athletic performance" OR "sport performance" OR "physical performance" OR "technical performance" OR "decision making" OR "reaction time") AND TS=(athlete OR athletes OR sport*) | 1121 |
| Embase | ('brain endurance training':ti,ab,kw OR 'cognitive endurance training':ti,ab,kw OR 'mental endurance training':ti,ab,kw) AND ('athletic performance':ti,ab,kw OR 'sport performance':ti,ab,kw OR 'physical performance':ti,ab,kw OR athlete*:ti,ab,kw OR sport*:ti,ab,kw) | 197 |
| SPORTDiscus | TX ("brain endurance training" OR "cognitive endurance training") | 17 |
| Cochrane Library | OR "mental endurance training") AND TX (performance OR athlete* OR sport OR endurance OR cognition) | 342 |

**Table S4 PEDro Scores of the Included Trials.**

| Author, year | D1 | D2 | D3 | D4 | D5 | D6 | D7 | D8 | D9 | D10 | D11 | Total |
| --- | --- | --- | --- | --- | --- | --- | --- | --- | --- | --- | --- | --- |
| Dalton de et al.2023 | Y | 1 | 0 | 1 | 0 | 0 | 0 | 1 | 1 | 1 | 1 | 6 |
| Jesús Díaz-García et al. 2023 | Y | 1 | 0 | 1 | 0 | 0 | 1 | 1 | 1 | 1 | 1 | 7 |
| Jesús Díaz-García et al. 2024 | Y | 1 | 0 | 1 | 0 | 0 | 0 | 1 | 1 | 1 | 1 | 6 |
| Jesús Díaz-García et al. 2025 | Y | 1 | 0 | 1 | 0 | 0 | 0 | 1 | 1 | 1 | 1 | 6 |
| Neil Dallaway et al. 2021 | Y | 1 | 0 | 1 | 0 | 0 | 0 | 1 | 1 | 1 | 1 | 6 |
| Neil Dallaway et al. 2023 | Y | 1 | 0 | 1 | 0 | 0 | 0 | 1 | 1 | 1 | 1 | 6 |
| Neil Dallaway et al. 2024 study1 | Y | 1 | 0 | 1 | 0 | 0 | 0 | 1 | 1 | 1 | 1 | 6 |
| Neil Dallaway et al. 2024 study2 | Y | 1 | 0 | 1 | 0 | 0 | 0 | 1 | 1 | 1 | 1 | 6 |
| Varesco et al.2025 | Y | 0 | 0 | 1 | 0 | 0 | 0 | 1 | 0 | 1 | 1 | 4 |
| Walter Staiano et al. 2022 | Y | 1 | 0 | 1 | 0 | 0 | 0 | 1 | 0 | 1 | 1 | 5 |
| Walter Staiano et al. 2023 study1 | Y | 1 | 0 | 1 | 0 | 0 | 0 | 1 | 0 | 1 | 1 | 5 |
| Walter Staiano et al. 2023 study2 | Y | 1 | 0 | 1 | 0 | 0 | 0 | 1 | 0 | 1 | 1 | 5 |
| Walter Staiano et al. 2025 | Y | 1 | 0 | 1 | 0 | 0 | 0 | 1 | 0 | 1 | 1 | 5 |

Note: studies scoring ≥6 are considered high quality, those scoring 4-5 are considered moderate quality, and those scoring ≤3 are considered low quality; Y, YES; N, NO.

D1. eligibility criteria were specified (not included in the total score)

D2. subjects were randomly allocated to groups (in a crossover study, subjects were randomly allocated an order in which treatments were received)

D3. allocation was concealed

D4. the groups were similar at baseline regarding the most important prognostic indicators

D5. there was blinding of all subjects

D6. there was blinding of all therapists who administered the therapy

D7. there was blinding of all assessors who measured at least one key outcome

D8. measures of at least one key outcome were obtained from more than 85% of the subjects initially allocated to groups

D9. all subjects for whom outcome measures were available received the treatment or control condition as allocated or, where this was not the case, data for at least one

key outcome was analysed by “intention to treat”

D10. the results of between-group statistical comparisons are reported for at least one key outcome

D11. the study provides both point measures and measures of variability for at least one key outcome

**Table S5. Exploratory Multilevel Egger-Type Regression Across Outcomes**

| Outcome | Egger Estimate | 95% CI Lower | 95% CI Upper | P value |
| --- | --- | --- | --- | --- |
| Cognitive Function | 12.62 | 2.31 | 22.94 | 0.034 |
| Physical Performance | 25.53 | 1.45 | 49.61 | 0.046 |
| Perceived Exertion | -3.92 | -16.39 | 8.56 | 0.220 |

**Table S6 Certainty of Evidence Assessed Using GRADE**

| Quality assessment | | | | | | | No of patients | | Effect | Quality | Importance |
| --- | --- | --- | --- | --- | --- | --- | --- | --- | --- | --- | --- |
|  |  |  |  |  |  |  |  |  |  |  |  |
| No of studies | **Design** | **Risk of bias** | **Inconsistency** | **Indirectness** | **Imprecision** | **Publication bias** | **BET** | **CON** | **Absolute** |  |  |
| Physical Performance (measured with: Hedge's; Better indicated by higher values) | | | | | | | | | | | |
| 12 | Randomised and non-randomised controlled trials | None | Serious^1^ | None | None | Serious^2^ | 205 | 200 | SMD 0.48 (0.34 to 0.62) | ÅOOO Low | CRITICAL |
| Cognitive Function (measured with: Hedge's; Better indicated by higher values) | | | | | | | | | | | |
| 11 | Randomised and non-randomised controlled trials | None | Serious^1^ | None | None | Serious^2^ | 193 | 188 | SMD 0.64 (0.24 to 1.04) | ÅOOO Low | CRITICAL |
| Perceived Exertion (Measured with: Hedges’ g; Better indicated by lower values) | | | | | | | | | | | |
| 8 | Randomised and non-randomised controlled trials | None | None | None | Serious^3^ | None | 108 | 106 | SMD -0.01 (-0.25 to 0.23) | ÅÅOO Moderate | CRITICAL |

^1^ Downgraded one level for inconsistency because substantial heterogeneity was observed (I² > 50%).
^2^ Downgraded one level for publication bias because Egger’s regression test indicated significant small-study effects (P < .05), suggesting potential publication bias.

^3^ Downgraded one level for imprecision because the 95% CI crossed the null and was compatible with both small negative and small positive effects.

**Table S7. Summary of Sensitivity Analyses for Cognitive Function, Physical Performance, and Perceived Exertion**

| **Cognitive Function** | | | | | | | |
| --- | --- | --- | --- | --- | --- | --- | --- |
| **Sensitivity analysis** | **Assumption / restriction** | **k** | **No. of studies** | **Hedges' g** | **95% CI** | **P value** | **I² total (%)** |
| Outlier exclusion | Before removal (r = 0.6) |  |  | 0.82 | [0.29, 1.34] | 0.007 | 90.9 |
| Outlier exclusion | After removal (r = 0.6) |  |  | 0.64 | [0.24, 1.04] | 0.006 | 82.4 |
| Within-study correlation | r = 0.4 (after outlier removal) |  |  | 0.65 | [0.25, 1.06] | 0.006 | 82.3 |
| Within-study correlation | r = 0.6 (after outlier removal) |  |  | 0.64 | [0.24, 1.04] | 0.006 | 82.4 |
| Within-study correlation | r = 0.8 (after outlier removal) |  |  | 0.64 | [0.24, 1.03] | 0.006 | 83.0 |
| Pre–post correlation | r = 0.4 |  |  | 0.65 | [0.26, 1.03] | 0.005 | 81.2 |
| Pre–post correlation | r = 0.6 |  |  | 0.75 | [0.28, 1.22] | 0.007 | 86.1 |
| Pre–post correlation | r = 0.8 |  |  | 0.98 | [0.34, 1.62] | 0.009 | 91.7 |
| Study-design restriction | Excluding non-RCT studies | 36 | 10 | 0.73 | [0.30, 1.16] | 0.005 |  |
| Control-condition restriction | Passive control only | 34 | 7 | 0.43 | [−0.05, 0.91] | 0.070 |  |
| **Physical Performance** | | | | | | | |
| **Sensitivity analysis** | **Assumption / restriction** | **k** | **No. of studies** | **Hedges' g** | **95% CI** | **P value** | **I² total (%)** |
| Outlier exclusion | Before removal (r = 0.6) |  |  | 0.44 | [0.09, 0.78] | 0.019 | 87.5 |
| Outlier exclusion | After removal (r = 0.6) |  |  | 0.48 | [0.34, 0.62] | <0.001 | 80.7 |
| Within-study correlation | r = 0.4 (after outlier removal) |  |  | 0.51 | [0.36, 0.66] | <0.001 | 79.9 |
| Within-study correlation | r = 0.6 (after outlier removal) |  |  | 0.48 | [0.34, 0.62] | <0.001 | 80.7 |
| Within-study correlation | r = 0.8 (after outlier removal) |  |  | 0.46 | [0.32, 0.59] | <0.001 | 81.5 |
| Pre–post correlation | r = 0.4 |  |  | 0.41 | [0.23, 0.58] | 0.001 | 78.2 |
| Pre–post correlation | r = 0.6 |  |  | 0.49 | [0.29, 0.69] | 0.001 | 83.9 |
| Pre–post correlation | r = 0.8 |  |  | 0.67 | [0.41, 0.93] | <0.001 | 90.4 |
| Study-design restriction | Excluding non-RCT studies | 86 | 11 | 0.47 | [0.31, 0.63] | <0.001 |  |
| Control-condition restriction | Passive control only | 81 | 8 | 0.44 | [0.28, 0.60] | 0.001 |  |
| **Perceived Exertion** | | | | | | | |
| **Sensitivity analysis** | **Assumption / restriction** | **k** | **No. of studies** | **Hedges' g** | **95% CI** | **P value** | **I² total (%)** |
| Outlier exclusion | Before removal (r = 0.6) |  |  | -0.16 | [−0.55, 0.22] | 0.347 | 47.6 |
| Outlier exclusion | After removal (r = 0.6) |  |  | -0.01 | [−0.25, 0.23] | 0.922 | 18.4 |
| Within-study correlation | r = 0.4 (after outlier removal) |  |  | -0.01 | [−0.25, 0.23] | 0.908 | 0.0 |
| Within-study correlation | r = 0.6 (after outlier removal) |  |  | -0.01 | [−0.25, 0.23] | 0.922 | 18.4 |
| Within-study correlation | r = 0.8 (after outlier removal) |  |  | -0.01 | [−0.25, 0.22] | 0.884 | 39.9 |
| Pre–post correlation | r = 0.4 |  |  | -0.02 | [−0.33, 0.30] | 0.905 | 0.0 |
| Pre–post correlation | r = 0.6 |  |  | -0.01 | [−0.37, 0.35] | 0.948 | 10.8 |
| Pre–post correlation | r = 0.8 |  |  | -0.03 | [−0.57, 0.51] | 0.896 | 58.8 |
| Study-design restriction | Excluding non-RCT studies | 14 | 7 | -0.01 | [−0.25, 0.23] | 0.920 |  |
| Control-condition restriction | Passive control only | 9 | 4 | 0.09 | [−0.33, 0.51] | 0.530 |  |

**Figure S1:** Risk of bias in randomized controlled trials (RoB 2).

**Figure S2:** Risk of bias in non-randomized studies (ROBINS-I).

**Figure S3 leave-one-out of cognitive function.**

**Figure S4 Leave-one-out of physical performance.**

**Figure S5 Leave-one-out of perceived exertion.**
